# Editorial: Non-insulin hypoglycemic drugs in type 2 diabetes remission

**DOI:** 10.3389/fendo.2023.1325071

**Published:** 2023-10-31

**Authors:** Kai-heng Guo, Xiao-ying Zhou, Chun-ping Zeng

**Affiliations:** Department of Endocrinology and Metabolism, The Fifth Affiliated Hospital of Guangzhou Medical University, Guangzhou, China

**Keywords:** diabetes, T2DM, diabetes remission, hypoglycemic drugs, glycemic control

In recent years, the global prevalence of diabetes is rising every year, with type 2 diabetes mellitus (T2DM) accounting for 96.0% of all diabetes cases in 2021. A study predicted that by 2050, more than 1.31 billion people worldwide will develop diabetes, with the rise in prevalence of diabetes is expected to be driven by the increase in T2DM (1). With the increasing economic burden of T2DM on families, countries and even the world, our goal is not only control and prevent T2DM, but also to achieve the type 2 diabetes remission. According to the American Diabetes Association (ADA), diabetes remission was defined as glycosylated hemoglobin (HbA1c) <6.5% after stopping glucose-lowering medication for at least 3 months (2). This Research Topic aims to address the challenge of diabetes by presenting and discussing strategies and recent advances in type 2 diabetes remission.

Based on individualized treatment needs, different approaches can be taken to achieve T2DM remission. Ricci et al. concluded that type 2 diabetes remission is achieved mainly through the following strategies: metabolic surgery, lifestyle interventions, and glucose-lowering medications. Finally they argued that weight loss surgery is the most effective measure to achieve and maintain long-term diabetes remission. It significantly improves glycemic control, reduces the need for hypoglycemic drugs and insulin, and can achieve diabetes remission in up to 60-80% of diabetes cases in the short term.

Intensive lifestyle intervention is a recommended as grade of A for achieving diabetes remission. Wei et al. reported that long-term T2DM remission could be achieved by very low caloric restriction (VLCR), and the median length of time to achieve remission after VLCR was 7.83 years, with a remission rate of 38.64%. They also explored duration of diabetes, fasting blood glucose (FBG), fasting insulin (FINS), HOMA-β, insulin area under curve (IAUC) in intravenous glucose tolerance test (IVGTT) and oral glucose tolerance test (OGTT) may be predictive factors associated with T2DM remission.

Type 2 diabetes remission through medication is achieved primarily through weight loss as well as reduction of islet load. Lam-Chung reported that SGLT2 inhibitors (SGLT2i) act on the kidneys through osmotic diuresis, which favor glucose extraction, thereby lowering blood glucose and HbA1c. However, since the overall effect stabilizes and then diminishes rapidly, the reduction of HbA1c is limited to the short term, making SGLT2i a “moderate” antidiabetic drug as defined by the European Medicines Agency (EMA). At the same time, the role of SGLT2i in reducing cardiovascular events and mortality was confirmed. Among them, empagliflozin was advantageous in reducing cardiovascular deaths, dapagliflozin was superior in reducing hospitalization for heart failure, and canagliflozin was beneficial in reducing cardiac events. The most recent version of the ADA guidelines this year added a new drug class, GLP-1/GIP dual agonists, involving the specific drug Tirzepatide, whose potent (weight loss and glucose lowering) effects bring new strategies for type 2 diabetes remission. Zeng et al. conducted a systematic review and meta-analysis of nine studies to explore the safety issues of tirzepatide in T2DM and obesity. They reported that no significant association was found between tirzepatide and increased risk of pancreatitis. The combined risk of gallbladder or biliary tract disease was significantly associated with tirzepatide, but not with the risk of cholelithiasis, cholecystitis, or biliary tract disease. Traditional Chinese medicine (TCM) can regulate the intestinal flora of T2DM patients, improve glucose metabolism, and reduce the risk of adverse effects caused by the western medicine, such as gastrointestinal and hypoglycemic reactions. Ma et al. conducted a network meta-analysis of 64 randomized controlled trials that evaluated the efficacy of TCM combined with conventional western medicine (CWM) for the treatment of T2DM, including FBG, 2-hour postprandial glucose (2hPG), HbA1c, adverse effects, and clinical efficacy. Finally, they reported that the clinical efficacy and safety of different TCM combined with CWM in treatment of T2DM were more significant compared with CWM alone, among which shenqi jiangtang granule combined with CWM had the best effect. The shenqi jiangtang granule combined with sulfonylurea, shenqi jiangtang granule combined with metformin and jinlida granule combined with insulin have significant effects on the reduction of FBG, 2hPG, and the clinical efficacy. Wang et al. addressed the mechanism of action of dihydromyricetin (DMY) for the treatment of T2DM. They reported that the manufacture of AMPK is a key target of DMY for the treatment of diabetes. DMY plays a hypoglycemic role in diabetes by improving glucose and lipid metabolism, attenuating inflammatory responses, and minimizing oxidative stress, with the signal transduction pathways underlying the regulation of AMPK or mTOR/autophagy, and relevant downstream cascades, including PGC-1α/SIRT3, MEK/ERK, and PI3K/Akt signal pathways. It is of great value for future development of relevant hypoglycemic drugs to achieve diabetes remission.

In conclusion, these articles provide new insights or strategies for achieving type 2 diabetes remission. Diabetes remission is an evolving and updated area that holds great promise for improving the quality of life for patients with diabetes. In addition, as the strategies for managing type 2 diabetes are constantly evolving, research on new pharmacologic therapies, lifestyle interventions, weight loss surgical modalities will require ongoing review and consideration.

## Author contributions

K-HG: Writing – original draft. X-YZ: Writing – original draft. C-PZ: Writing – review & editing.
